# Dissecting the genetic switches controlling salt tolerance in bermudagrass (*Cynodon dactylon*)

**DOI:** 10.1093/plcell/koag055

**Published:** 2026-03-02

**Authors:** Ju-Chen Chia

**Affiliations:** Assistant Features Editor, The Plant Cell, American Society of Plant Biologists; Plant Biology Section, School of Integrative Plant Science, Cornell University, Ithaca, NY, United States

Soil salinization, largely caused by the accumulation of sodium chloride, is one of the greatest challenges in agriculture. Excess salt restricts water uptake and leads to ion overaccumulation in plant tissues, thereby attenuating growth and productivity (Munns and Tester 2008). To limit ion accumulation under saline conditions, plants strongly activate the Salt Overly Sensitive (SOS) pathway, including a key Na^+^/H^+^ antiporter, SOS1, that actively exports Na^+^ across the plasma membrane in response to salinity (Shi et al. 2002). Although the core components of the SOS pathway have been well characterized in model species (Ali et al. 2023), they remain poorly understood in turfgrasses and forage crops. Bermudagrass (Cynodon dactylon) exhibits exceptional salt tolerance and natural genetic variation, making it a powerful system for studying salt stress responses in economically valuable grasses (Cui et al. 2025).

In recent work, **Shurui Song and colleagues** (Song et al. 2026) characterized bermudagrass *SOS1* (*CdSOS1*) and its upstream regulatory network mediating salt tolerance through both transcriptional and post-translational regulation. The authors found that *CdSOS1* was strongly upregulated in salt-tolerant germplasms under salinity, and that *CdSOS1* knockdown markedly increased electrolyte leakage, Na^+^ accumulation, and the Na^+^/K^+^ ratio, underscoring its essential role in salt resilience. Using a yeast one-hybrid (Y1H) screen with the *CdSOS1* promoter sequence as bait, the authors identified a MYB transcription factor, CdMYB5, that directly binds the *CdSOS1* promoter to activate its transcription. Elevated *CdMYB5* expression significantly increased salt tolerance and *CdSOS1* expression in bermudagrass. Importantly, silencing *CdSOS1* in *CdMYB5*-overexpression plants reduced the enhanced tolerance, demonstrating that CdMYB5 acts upstream of *CdSOS1* to confer salt tolerance.

Because *CdMYB5* is transcriptionally regulated under salt stress, the authors searched for its upstream regulators using a Y1H screen with the *CdMYB5* promoter as bait and identified CdMYB26, which acts as a suppressor of *CdMYB5* transcription. Accordingly, *CdMYB5* expression was strongly reduced in *CdMYB26*-overexpression lines and markedly elevated in *CdMYB26*-silenced plants. Consistent with its role as a *CdMYB5* repressor, *CdMYB26* expression in these transgenic lines was negatively correlated with salt tolerance traits.

Notably, although *CdMYB26*, *CdMYB5*, and *CdSOS1* were all transcriptionally upregulated by salt stress, only the transcript abundance of *CdMYB5* and *CdSOS1*, but not *CdMYB26*, aligned with salinity-tolerance phenotypes across bermudagrass germplasms. This finding suggests that *CdMYB26* is regulated post-transcriptionally. In line with this hypothesis, the authors found that the CdMYB26-GFP fusion protein overexpressed in bermudagrass underwent salinity-induced degradation, and that treatment with the proteasome inhibitor MG132 significantly stabilized the protein. The authors further showed that CdMYB26 directly interacts with an F-box protein, CdFBX1 (Cd8B1G007220). Because F-box proteins act as substrate-recognition components of SCF ubiquitin-protein ligases, this interaction supports a model in which proteasome-mediated protein turnover regulates CdMYB26 activity.

Interestingly, 2 variants of the *CdFBX1* coding sequence were identified across bermudagrass germplasms: *CdFBX1.1*, which encodes the full-length protein, and *CdFBX1.2*, which encodes a truncated isoform resulting from early translation termination. Germplasms harboring *CdFBX1.2* exhibited greater salt tolerance and higher expression of the *CdMYB5*-*CdSOS1* pathway than those containing *CdFBX1.1*. Although both CdFBX1 isoforms interacted with CdMYB26 in co-immunoprecipitation assays, CdFBX1.2 bound CdMYB26 with higher affinity, and only CdFBX1.2 promoted CdMYB26 ubiquitination and subsequent proteasome degradation. These results highlight CdFBX1.2 as the functional variant contributing to salt tolerance.

In summary, Song et al. identified the CdFBX1-CdMYB26-CdMYB5 regulatory module as a key pathway controlling salt adaptation in bermudagrass by fine-tuning *CdSOS1* expression. Under salinity, CdFBX1.2 interacts with CdMYB26 and targets it for proteasome-mediated degradation, thereby relieving repression of *CdMYB5* transcription and ultimately activating *CdSOS1* (see Fig. 1). The authors further highlight *CdFBX1.2* as a promising genetic marker for breeding salt-tolerant forage crops. Moreover, the distinct activities of CdFBX1.1 and CdFBX1.2 provide a valuable framework for exploring how specific F-box domains direct MYB ubiquitination during salt stress.

**Figure 1:**
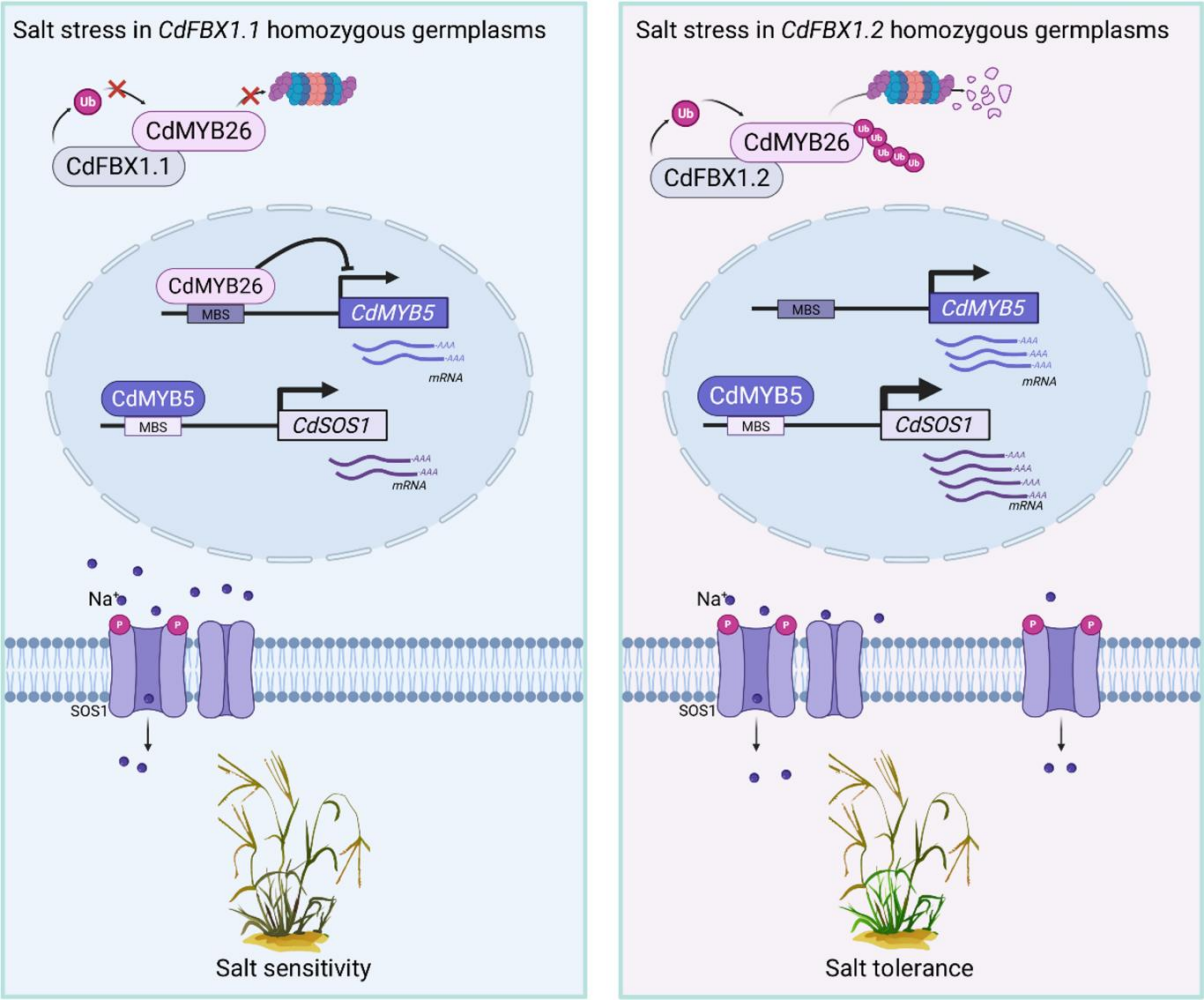
A model illustrating the CdFBX1-CdMYB26-CdMYB5 regulatory module controlling *CdSOS1* expression in bermudagrass. Under salt stress, *CdMYB5* is upregulated and promotes *CdSOS1* expression, whereas CdMYB26 represses *CdMYB5* transcription. Only the CdFBX1.2 isoform facilitates CdMYB26 ubiquitination and proteasomal degradation, thereby activating the CdMYB5-CdSOS1 pathway. Adapted from Song et al. (2026), Figure 10.

## Recent related articles in *The Plant Cell:*

- Lu et al. (2023) showed that CycC1;1, a C-type cyclin subunit of the plant Mediator complex, negatively regulates salt tolerance by suppressing WRKY75-dependent *SOS1* transcription in *Arabidopsis thaliana*.
- Chen et al. (2024) reported that OsFBO13, an F-box–containing protein in rice, negatively regulates salt tolerance and grain size by facilitating the ubiquitination and proteasomal degradation of OsGRF7, which confers salinity resistance through arbutin biosynthesis.
- Chen et al. (2025) identified *OsMYB39a*, *OsMYB41*, *OsMYB92a*, and *OsMYB92b* as core regulators of endodermal suberization and lignification in rice, acting downstream of stress-inducible signaling to promote suberin accumulation and enhance salt adaptation.

## Data Availability

No new data were generated or analysed in support of this research.
